# Dose-response relation of self-reported and accelerometer-measured physical activity to perceived health in middle age—the Northern Finland Birth Cohort 1966 Study

**DOI:** 10.1186/s12889-018-6359-8

**Published:** 2019-01-07

**Authors:** Maisa S. Niemelä, Maarit Kangas, Riikka J. Ahola, Juha P. Auvinen, Anna-Maiju Leinonen, Tuija H. Tammelin, Eeva S. Vaaramo, Sirkka M. Keinänen-Kiukaanniemi, Raija I. Korpelainen, Timo J. Jämsä

**Affiliations:** 1 Research Unit of Medical Imaging, Physics and Technology, University of Oulu, PO Box 5000, 90014 Oulu, Finland; 2 Infotech, University of Oulu, Oulu, Finland; 3 Medical Research Center, Oulu University Hospital and University of Oulu, Oulu, Finland; 4 Polar Electro, Kempele, Finland; 5 Center for Life Course Health Research, University of Oulu, Oulu, Finland; 6 Department of Sports and Exercise Medicine, Oulu Deaconess Institute, Oulu, Finland; 7 LIKES Research Centre for Physical Activity and Health, Jyväskylä, Finland; 8 Health Center of Oulu, Oulu, Finland; 9 Healthcare and Social Services of Selänne, Pyhäjärvi, Finland; 10 Diagnostic Radiology, Oulu University Hospital, Oulu, Finland

**Keywords:** Physical exercise, Questionnaires, Self-rated health, Middle aged

## Abstract

**Background:**

Regular physical activity (PA) promotes health and decreases mortality. The positive relationship between PA and perceived health (PH) is well known. However, previous research in the field has often used self-reported PA measures. The aim of this population-based NFBC1966 birth cohort study was to assess the relationship between both self-reported and objectively measured PA and PH in midlife.

**Methods:**

A sample group of 6384 participants (2878 men, 3506 women, response rate 62%) aged 46 completed a questionnaire on PH and health behaviors, including items on weekly leisure time physical activity (LTPA) and daily sitting time (ST). PH was dichotomized as good (very good or good) and other (fair, poor, or very poor). PA was measured with a wrist-worn Polar Active (Polar Electro, Finland) accelerometer for 14 days (*n* = 5481, 98%) and expressed as daily average time spent in moderate to vigorous intensity PA (MVPA). Odds ratios (OR) and 95% confidence intervals for good PH were calculated using binary logistic regression and adjusted for relevant demographic, socioeconomic, and health characteristics, and ST.

**Results:**

The level of PA was positively associated with PH after adjustments with covariates and ST. There was a dose-response relationship across the PA quartiles according to the adjusted multivariable models. Self-reported LTPA was more strongly associated with good PH (OR from 1.72 to 4.33 compared to lowest PA quartile) than objectively measured PA (OR from 1.37 to 1.66 compared to lowest PA quartile).

**Conclusions:**

In this large population-based birth cohort study, we for the first time show a positive dose-response relationship of both self-reported and objectively measured PA to PH, the relationship being stronger for self-reported LTPA. Despite the cross-sectional design of this study, the results from this large sample suggest that both self-reported and objectively measured physical activity are strongly associated with PH, which is a predictor of morbidity and mortality, and regular PA should be encouraged in midlife.

## Background

Perceived health (PH; also known as self-rated health and self-perceived health) is a widely used, inexpensive health measure. Most often a single-item question asking individuals to rate their current health status on a 4- or 5-point scale from *poor* to *excellent* is used. PH is a subjective health measure that includes physical, mental, emotional, and functional aspects of health [1]. PH has been shown to be associated with mortality, morbidity, and objective health status [2, 3] and to predict mortality after controlling for objective health status [2]. In some cases, it has been an even stronger predictor of survival than medical records [4].

Lifestyle, social, and work-related factors have been identified as determinants of PH [3, 5, 6], and it has been acknowledged that people with similar health conditions may provide different answers to a PH question [7]. Thus, PH includes components beyond objective health status, as Maddox and Douglass noted decades ago: “These ratings clearly measure something more—and something less—than objective medical rating” [8].

It has been widely established that sufficient and regular physical activity (PA) enhances health and decreases the risk of morbidity and mortality. The lack of physical activity, or physical inactivity, increases the risk of many non-communicable diseases and shortens the lifespan over three years [9, 10], and the global cost of physical inactivity was evaluated as more than 53 billion international dollars in 2013 [11].

Recently, objective wearable measuring devices such as accelerometers and pedometers have become popular in the PA research field [12]. They provide accurate information about PA due to the lack of common errors (recall, and social desirability bias) inherent in self-reports of PA [13]. However, both measuring conditions may cause reactivity, i.e. changes in habitual PA behavior during the measurement period.

Self-reported leisure-time PA (LTPA) [14, 15] and moderate to vigorous PA (MVPA) measured with an accelerometer [16–18] have both separately been reported to be associated with PH in many age groups. However, previous research on the determinants of PH has often relied on self-reported PA, and questions have been raised about the potential conceptual overlap between self-reported PA and health due to the complex nature of human perception of health and well-being, including physical, social, and mental constructs [16]. To our knowledge, few studies have compared objective and subjective PA measures in relation to subjective health measures. Anokye et al. reported a stronger relationship between objective PA and health-related quality of life (HRQoL) compared to subjectively measured PA [19].

Sedentary time increases in adulthood with age, and those participating in LTPA or occupational PA in early middle age are more likely to engage in LTPA as they age [20, 21]. Additionally, a connection between higher age and poorer PH has been recognized [22]. Therefore, reaching a sufficient level of PA in middle age, and maintaining this level while aging, is a key factor for healthy aging. Thus, the aim of the study was to determine and compare the relationship between self-reported and accelerometer-measured PA and PH in midlife, in the context of a wide population-based birth cohort study from Northern Finland. To our knowledge, this is the first study exploring the relationship between both measured and self-reported PA and PH in a single study setup. We hypothesized that both PA variables have a positive association with PH and that objectively measured PA might have a stronger association compared to self-reported PA due to its ubiquitous nature including all measured activities around the clock.

## Methods

### Study population

The Northern Finland Birth Cohort 1966 study (NFBC1966) includes subjects in Northern Finland whose dates of birth fell in the year 1966 (*n* = 12,058 live births) [23]. Information about these individuals has been recorded regularly since their birth through health care records, questionnaires, and clinical examinations, and data has been collected on their parents and offspring. The study was approved by the Ethical Committee of the Northern Ostrobothnia Hospital District in Oulu, Finland (94/2011), and it has been performed in accordance with the Declaration of Helsinki. The subjects and their parents provided written consent for the study. Personal identity information was encrypted and replaced with identification codes to provide full anonymity. This study analyzed the data obtained from the most recent time point, at which time participants were 46 years old (*n* = 10,321). Participants attended clinical examinations (*n* = 5852), where trained nurses thoroughly assessed their medical condition, their height and weight were measured, and their BMI (body mass index) calculated.

### Questionnaire

Postal questionnaires were sent to all participants with known addresses in 2012–2014 (PA questionnaire response rate 62%, *n* = 6384). Questionnaires included items about participants’ health, behavior, and social background. Their marital status, education, employment status, annual household income, and prevalence of diagnosed diseases were addressed. Smoking status (former, current, non-smoker) and alcohol consumption (g/day) were composed based on multiple questions about drinking and smoking habits.

Participants were asked about their PH with the question “How would you describe your health at the moment?” The response alternatives were 1) very good, 2) good, 3) fair, 4) poor, and 5) very poor. The PH responses were dichotomized as good (very good and good) and other (fair, poor, and very poor) [2].

LTPA was self-reported with questions on the frequency and duration of light and brisk physical activities or exercises during leisure time, as was done in the 31-years follow-up study (24). Brisk PA was described as causing at least some sweating and breathlessness, while light PA was defined as causing no sweating or breathlessness. PA frequency had six response options: 1) once a month or less often, 2) 2–3 times a month, 3) once a week, 4) 2–3 times a week, 5) 4–6 times a week, and 6) daily. PA duration had the following response options: 1) not at all, 2) less than 20 min, 3) 20–39 min, 4) 40–59 min 5) 1–1.5 h, and 6) more than 1.5 h [24]. Weekly averages of metabolic equivalent of a task (MET) minutes of light and brisk PA were calculated by multiplying the PA volume (duration*frequency) by its intensity (light PA 3 METs and brisk PA 5 METs) [25].

Daily sitting time (ST) was assessed with the question “How much time do you spend sitting on a normal weekday?” The response was divided to describe the amount of sitting in five domains (at work, at home watching TV or video, at home in front of computer, in a vehicle, and in another place) [26]. Average daily ST (min/day) was calculated as a sum of durations of these sedentary behaviors. Those reporting ST higher than 18 h/day (*n* = 27) were excluded from analyses concerning ST.

### Accelerometer-measured physical activity

**Fig. 1:**
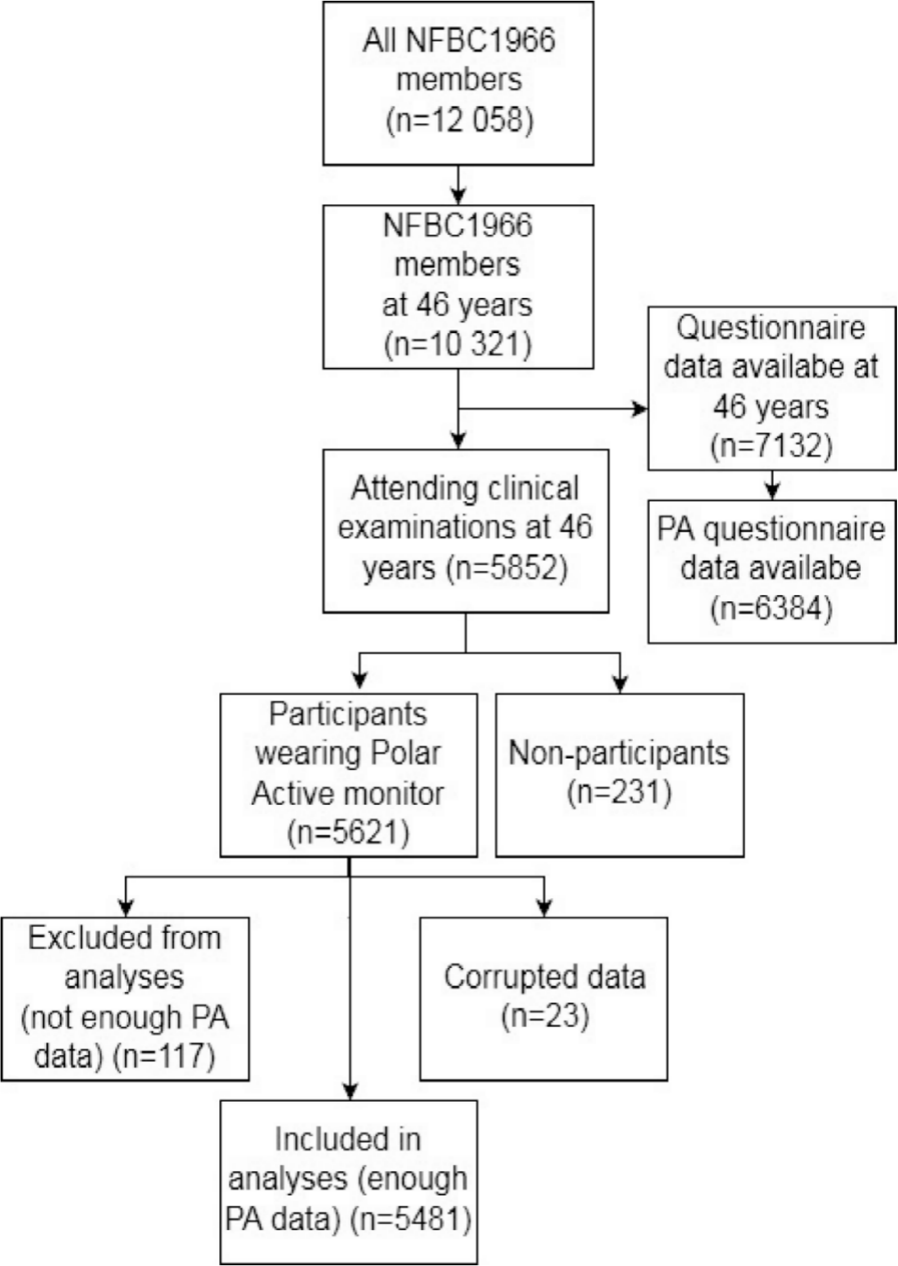
Northern Finland Birth Cohort 1966 (NFBC1966) participants

PA was objectively measured with wrist-worn activity monitors (Polar Electro Oy, Kempele, Finland) for 14 days. Polar Active is a waterproof accelerometer providing MET values every 30 s based on daily PA [27]. Polar Active has been shown to correlate (R^2^ = 0.74) with the double-labelled water technique when measuring energy expenditure during exercise [28]. It uses height, weight, gender, and age of the user as predefined inputs. Each activity monitor was blinded, giving no feedback to the user, and the monitors were given to the participants during clinical examinations, with participants being instructed to mail them back after the measurement period. Participants were asked to wear the Polar Active monitor 24 h/day for at least 14 days on the non-dominant hand. Measured PA was classified as five levels (very light: 1–2 MET, light: 2–3.5 MET, moderate: 3.5–5 MET, vigorous: 5–8 MET, and vigorous+ ≥8 MET) [29]. Daily averages of duration spent in activity levels (min/day) were calculated for all participants. All activity performed with the intensity of 3.5 MET or higher was assessed as MVPA (min/day), and MVPA MET-minutes were calculated by multiplying each MET value with its duration (30s). The first day when the activity monitor was given to the participant was excluded from the analysis. Participants with four or more eligible days (wear time of at least 600 min/day) were included in the analyses (Fig. 1) [30].

### Statistics

The descriptive data is presented as numbers and percentages, means, standard deviations (SD) or medians, and 25th and 75th percentiles for skewed data. Accelerometer-measured moderate, vigorous, and very vigorous intensity PA and MVPA were natural log-transformed to obtain normal distribution. Non-transformed values are presented in the tables. Univariate associations between continuous variables and gender and PH were analyzed using the independent-samples t-test, with Tukey post hoc tests for normally distributed variables and with the independent-samples Mann-Whitney U test for skewed data. The agreement between objectively measured MVPA and questionnaire based LTPA as MET-minutes was evaluated with Spearman’s rank correlation coefficient (r). The associations of demographic, socioeconomic, and health characteristics to PH were studied through binary logistic regression analysis. PH was controlled for gender, marital status (married/cohabiting, unmarried), employment status (working, unemployed, other), education (vocational or no vocational education, polytechnic/university degree), prevalence of diagnosed diseases (cardiovascular disease, diabetes mellitus, cancer, musculoskeletal diseases, or mental disorder), smoking (former, current, non-smoker), heavy alcohol consumption (men ≥40 g/day, women ≥20 g/day), BMI (body mass index kg/m^2^, as continuous variable), income (quartiles of yearly household income), and ST (min/day, quartiles). Model 1 included in addition accelerometer-measured MVPA (min/week, quartiles), while Model 2 included self-reported LTPA (min/week, quartiles) instead of MVPA. Odds ratios (OR) and 95% confidence intervals (95% CI) for good PH were calculated, and ORs were adjusted for all the variables. The significance of the models was evaluated using the likelihood ratio test (χ^2^) and Nagelkerke R^2^ value. Statistical significance was set to *p* < 0.05, and statistical analyses were performed with IBM SPSS Statistics for Windows, version 24.0 (IBM Corp., Armonk, USA).

## Results

The study population consisted of 3291 (46%) men and 3841 (54%) women (Table 1).

**Table 1:** Characteristics of the study population (*n* = 7132)

Variable	Men (*n* = 3291)	Women (*n* = 3841)	All (*n* = 7132)
Height, cm	178.5 (6.3)	164.8 (6.0)	170.8 (9.1)
Weight, kg	87.2 (14.9)	72.0 (14.9)	78.7 (16.7)
BMI, body mass index	27.3 (4.3)	26.5 (5.3)	26.9 (4.9)
Education			
No professional education	148 (5%)	106 (3%)	254 (4%)
Vocational/college level education	2174 (72%)	2339 (66%)	4513 (68%)
Polytechnic/university degree	699 (23%)	1125 (31%)	1824 (28%)
Employment status			
Employed	2571 (87%)	3051 (87%)	5622 (87%)
Studying	33 (1%)	68 (2%)	101 (2%)
Unemployed	220 (8%)	182 (5%)	402 (6%)
Other	122 (4%)	207 (6%)	329 (5%)
Smoking			
Non-smoker	1366 (45%)	2086 (57%)	3452 (51%)
Current smoker	762 (25%)	697 (19%)	1459 (22%)
Former smoker	923 (30%)	872 (24%)	1795 (27%)
Alcohol consumption			
Alcohol consumption, g/day	8.5 (2.4–21.8)	2.9 (0.6–8.1)	4.7 (1.0–14.0)
Heavy users^*^	330 (11%)	291 (8%)	621 (9%)

values are numbers (%), mean (SD), or median (25th –75th percentile), ^*^Heavy users men≥40 g/day, women≥20 g/day

The PA questionnaire was completed by 6384 participants, and ST questions were answered by 6735 participants. Self-reported LTPA and corresponding MET-minutes and self-reported STs are presented in Table 2. The weekly median of self-reported LTPA (total activity) was 225 min/wk., which corresponds to a total amount of 788 MET-min/wk. Women reported significantly more PA than men (*p* < 0.001). Self-reported overall median ST was 450 (300–600) min/day. Men reported 60 min more sitting per day than women (p < 0.05). Most of the time spent sitting took place at work and at home watching TV or video (Table 2).

**Table 2:** Self-reported leisure time physical activity (PA; min/wk) and sitting time (ST; min/day)

PA and ST type	Men^a^	Women^a^	All^a^
Total PA (min/wk)	188 (75–345)^*^	233 (115–398)	225 (90–375)
Light PA (min/wk)	75 (30–210)^*^	113 (50–225)	113 (45–210)
Brisk PA (min/wk)	75 (10–188)^*^	75 (23–188)	75 (15–188)
Total PA (MET-min/wk)	713 (248–1350)^*^	855 (435–1500)	788 (938–1463)
Total sitting time (min/d)	480 (315–615)^**^	420 (270–570)	450 (300–600)
Sitting at work (min/d)	210 (60–360)^**^	210 (60–390)	210 (60–390)
Sitting at home watching TV or video (min/d)	120 (60–150)^**^	120 (60–120)	120 (60–120)
Sitting at home in front of computer (min/d)	60 (30–60)^**^	30 (30–60)	60 (30–60)
Sitting in a vehicle (min/d)	60 (30–90)^**^	30 (15–60)	40 (30–60)
Sitting in an another place (min/d)	0 (0–60)^**^	0 (0–60)	0 (0–60)

median (25th–75th percentiles), ^a^for PA: men (*n* = 2878), women (*n* = 3506), all (n = 6384), for ST: men (*n* = 3681), women (*n* = 3054), all (*n* = 6735), ^*^different from women (p < 0.001), ^**^different from women (p < 0.05)

PA was measured by accelerometer, with 5621 participants from which eligible activity data was available from 5481 (98%). In addition, for some participants, the data had been corrupted and could not be restored due to activity monitors malfunctioning (*n* = 23). Those who provided eligible activity data were more often employed (*p* = 0.02) than the participants who did not have enough PA data (data not shown). For those with eligible PA data, the average measurement period was 13 days and average wearing time during waking hours was 978 (62) min/day. The weekly average of accelerometer-measured MVPA was 483 min/wk. (2590 MET-min/wk). Men had on average more MVPA and very light activity and less light activity compared to women (*p* < 0.001). The correlation between accelerometer-measured MVPA and self-reported LTPA MET-minutes was low (*r* = 0.296, p < 0.001). The mean daily time spent on different PA levels are presented in Table 3.

**Table 3:** Daily minutes of accelerometer-measured physical activity (PA)

Accelerometer measured PA	Men (*N* = 2413)	Women (*N* = 3068)	All (*N* = 5481)
Very light intensity PA (1–2 MET) (min/d)	644 (95)^*^	621 (88)	631 (92)
Light intensity PA (2–3.5 MET) (min/d)	266 (70)^*^	288 (73)	278 (72)
Moderate intensity PA (3.5–5 MET) (min/d)	48 (25)^*^	28 (15)	37 (22)
Vigorous intensity PA (5–8 MET) (min/d)	18 (13)^*^	28 (17)	23 (16)
Very vigorous intensity PA (> 8 MET) (min/d)	12 (13)^*^	5 (7)	8 (10)
MVPA (≥ 3.5 MET) (min/d)	79 (39)^*^	61 (29)	69 (35)
*MVPA MET-minutes (MET-min/d)*	422 (234)^*^	329 (177)	370 (209)

mean (SD), ^*^different from women (p < 0.001), MVPA = Moderate to Vigorous Physical Activity

Over half the study population (53%) perceived their health as good, 13% as very good, and 30, 3, and 1% as fair, poor, and very poor, respectively. The amount of accelerometer measured MVPA and self-reported LTPA was significantly higher among those who rated their health as at least good compared to those who rated it less than good (p < 0.001; Table 4). There was no significant difference between self-reported ST in those with good PH compared to others (*p* = 0.25).

**Table 4:** Self-reported leisure time physical activity (LTPA) and accelerometer-measured moderate-to-vigorous activity (MVPA) in relation to PH

	Good or very good PH			Fair, poor or very poor PH		
	Men (*n* = 1383)	Women (*n* = 1924)	All (*n* = 3307)	Men (*n* = 709)	Women (*n* = 877)	All (*n* = 1586)
MVPA (min/wk)	574 (256)	445 (201)	499 (234)^*^	507 (294)	385 (211)	440 (258)
LTPA (min/wk)^a^	233 (118–413)	263 (150–420)	255 (143–413)^*^	120 (30–120)	158 (68–300)	143 (45–270)
ST (min/day)^a^	482 (191)	435 (185)	455 (190)	481 (205)	441 (197)	459 (201)

mean (SD) or median (25th–75th percentiles), ^*^different from fair, poor or very poor (p < 0.001), ^a^self-reported, PH = perceived health

Both multivariate logistic regression models (Table 5) were statistically significant at the level of p < 0.001 in the likelihood ratio test (χ^2^ = 704.35 and χ^2^ = 882.19 for Models 1 and 2, respectively). The Nagelkerke R^2^ values were 0.212 and 0.262 for Models 1 and 2, respectively. MVPA (Model 1) was positively associated with good PH after adjustment of gender, BMI, prevalence of diseases, smoking, alcohol consumption, ST, and socioeconomic factors. MVPA ORs were greater (ORs = 1.37, 1.49, and 1.66) for the second, third, and fourth quartiles, respectively, compared to the lowest one. Furthermore, greater LTPA (Model 2) was associated with higher odds of good PH (ORs = 1.72, 2.41, and 4.33) for the second, third, and fourth quartiles respectively (Table 5). University or polytechnic degree, higher income (two highest quartiles), lack of diagnosed diseases, and marriage/cohabitation increased the odds of good PH. In contrast, heavy alcohol consumption, smoking, and higher BMI were associated with lower odds of good PH. ST and gender were not associated with PH.

**Table 5:** Odds ratios for good perceived health. Model 1 includes accelerometer measured MVPA, Model 2 self-reported LTPA

Variable	Model 1. N	OR (95% CI)^a^	Model 2. N	OR (95% CI)^a^
Gender				
Men	1931	1	1892	1
Women	2391	1.13 (0.97–1.31)	2380	0.96 (0.82–1.11)
Marital status				
Married/cohabiting	3459	1	3411	1
Unmarried	863	0.86 (0.71–1.05)	861	**0.77 (0.63–0.95)**
Education				
Vocational/college level education or no vocational education	3021	1	2987	1
Polytechnic/university degree	1301	**1.31 (1.10–1.56)**	1285	**1.28 (1.08–1.53)**
Employment status				
Working	3893	1	3837	1
Unemployed	218	**0.54 (0.40–0.74)**	220	**0.48 (0.35–0.65)**
Other	211	**0.44 (0.32–0.60)**	215	**0.39 (0.28–0.53)**
Smoking				
Non-smoker	2341	1	2315	1
Current smoker	782	**0.60 (0.50–0.73)**	783	**0.64 (0.53–0.78)**
Former smoker	1199	1.00 (0.84–1.18)	1174	0.97 (0.82–1.15)
Prevalence of diseases^b^				
One or more	2960	1	2935	1
No	1362	**2.16 (1.83–2.55)**	1337	**2.27 (1.92–2.69)**
Heavy alcohol consumption^c^				
Yes	353	1	364	1
No	3969	**1.43 (1.12–1.83)**	3908	**1.31 (1.02–1.69)**
BMI (continuous, kg/m2)	4322	**0.90 (0.89–0.91)**	4272	**0.90 (0.89–0.92)**
Income quartiles (k€/year)				
I (0–34.5)	936	1	941	1
II (34.6–59.5)	1109	1.14 (0.93–1.40)	1097	1.11 (0.90–1.37)
III (59.6–79.9)	996	**1.51 (1.20–1.90)**	974	**1.43 (1.13–1.81)**
IV (≥80)	1281	**2.06 (1.62–2.62)**	1260	**1.84 (1.44–2.35)**
Sitting time quartiles (min/day)				
I (20–300)	982	1	958	1
II (301–480)	981	0.96 (0.78–1.18)	968	0.97 (0.78–1.20)
III (481–600)	1154	1.04 (0.84–1.23)	1160	1.06 (0.86–1.31)
IV (601–1080)	1205	0.87 (0.71–1.07)	1186	0.93 (0.75–1.14)
MVPA quartiles (min/week)				
I (7–313)	1049	1		
II (314–440)	1068	**1.37 (1.13–1.67)**		
III (441–603)	1091	**1.49 (1.21–1.82)**		
IV (604–2638)	1114	**1.66 (1.35–2.05)**		
LTPA quartiles (min/week)				
I (0–89)			914	1
II (90–224)			1167	**1.72 (1.41–2.09)**
III (225–374)			1060	**2.41 (1.96–2.96)**
IV (375–1260)			1131	**4.33 (3.47–5.40)**

*MVPA* Moderate to Vigorous Physical Activity, *LTPA* Leisure Time Physical Activity

^a^Statistically significant associations (p < 0.05) are bolded

^b^Prevalence of cardiovascular disease, diabetes mellitus, cancer, musculoskeletal diseases or mental disorder

^c^Alcohol consumption for men at least 40 g/day and for women at least 20 g/day

## Discussion

The aim of this population-based study was to investigate and compare the relationship of self-reported and accelerometer-measured physical activity to PH in midlife. The main finding was that both self-reported LTPA and accelerometer-measured MVPA were significantly and positively associated with PH. To our knowledge, this study for the first time reports the dose-response association between both self-reported and accelerometer-measured PA and PH in a large cross-sectional setup.

In the present study, the amount of accelerometer measured MVPA and self-reported LTPA was higher among those who perceived their health as at least good compared to those with fair, poor, or very poor health. When adjusted for socioeconomic, demographic, and health factors and ST, both MVPA and LTPA were positively associated with PH. The relationship was consistently stronger between LTPA and PH compared to measured MVPA, contrary to our hypothesis. Odds of good PH was over fourfold higher for those in the highest LTPA quartile compared to the lowest, as opposed to MVPA, where the odds of good PH were modestly higher in the highest quartile, around 1.7-fold, compared to the lowest quartile.

A similar framework was used in an earlier study involving objective and subjective measures of PA and health-related quality of life among middle aged participants [19]. However, the association between accelerometer-measured PA and HRQoL was stronger compared to questionnaire-based measurements of PA; the opposite was observed in our study, where LTPA had a stronger association with PH than objectively measured MVPA. This discrepancy might be due to different outcome measures (PH vs. HRQoL). Moreover, the possibility that PH and self-reported PA might have a conceptual overlap could explain the stronger relationship between them in this study. It has been suggested that especially LTPA would have stronger positive association with PH compared to other PA domains (like household or work related PA) [15, 31, 32]. LTPA is autonomous and often aims for recreation, enjoyment and social interaction and thus has potential to increase mood and psychological health in addition to physiological health benefits [31, 32]. In the study, the agreement between objectively MVPA and self-reported LTPA was low, which indicates that LTPA neglects other domains of PA (e.g. work related, commuting, and household activities) which can have moderate or higher intensity and thus are included in the objectively measured MVPA time.

Our findings are in line with an earlier study indicating a positive connection between PH and objectively measured PA and a lack of connection between PH and ST [16]. Previously, the dose-response relationship between LTPA and PH has also been acknowledged [14]. Other covariates for good PH found in this study, including higher education, marriage/cohabitation, higher income, and working status have also been widely acknowledged previously (see, e.g., [2, 3, 22]). In this study, gender was not related to PH.

Our results indicate that middle aged participants engaged in an average of 69 min of MVPA per day (2590 MET-min/wk), as measured with the activity monitor, with men participating in significantly more MVPA than women. In previous studies among middle-aged populations, the amount of accelerometer-measured PA has varied considerably. In the United States, MVPA duration was only 35 and 20 min/day in men and women respectively [33]. However, a study in Norway reported almost twice as much daily MVPA as the current study, with 117 min/day [34]. These studies relied on waist-worn monitors, and it has been found that wrist-worn monitors provide higher estimates of activity compared to waist-worn monitors due to the wear location [35]. Furthermore, using objective PA measurements may have effected on the PA behavior of the participants during the two-week measurement period in this study.

Self-reported time spent in light and brisk activity during leisure time in this study was around 3 h/wk., which is less than that reported in previous US study involving the same age group [36]. In this study, women reported significantly more LTPA than men, although based on the objective measurement, men engaged in more MVPA than women. The disparity between measured and self-estimated PA can be explained partly by the questionnaire addressing only the amount and duration of leisure time brisk and light activity. It has been established that 500–1000 MET-min/wk. of PA is enough to bring major health benefits [37]. With this criterion, the study population was sufficiently active based on both the questionnaire and the accelerometer.

Time spent sitting was on average 7.5 h/day based on self-report. Men reported an hour more ST than women per day. Most of the ST was reported to take place at work. Earlier studies involving accelerometer measurements show STs of around the same magnitude in middle-aged people: around 7.5 h/day in the United States and 8.5 h/day in Australia [21, 38].

This study has some limitations. Causality between PA and PH cannot be concluded due to the cross-sectional study design. The lack of accelerometer-measured ST is another limitation, as accelerometer measured and self-reported STs differ. Objective measurement is known to measure a greater amount of ST compared to self-reports [34]. Thus, the actual sitting time in this study might be underestimated. It has been established that measuring ST with wrist-worn monitor has limitations due to its difficulty in posture recognition and with methods used in this study it wasn’t possible to differentiate reliably sitting from other low intensity still activities, e.g. standing [39]. The amount of reported sitting time did not differ between good and poor PH, indicating that the amount of sitting is more weakly associated with perception of health compared to PA, which was clearly positively connected with PH.

The methodological limitations of objective and subjective measures of PA when investigating the relationships between PH and well-being should be acknowledged. While accelerometer measurement is a reliable and continuous measuring method, it does not capture all types of PA precisely, underestimating, for example, cycling and carrying loads. In contrast, PA questionnaires tend to overestimate the true PA level but can provide essential information about the PA type and context [36]. The strengths of the study include a representative population sample and high compliance of activity monitor wear.

## Conclusions

In this population-based study on middle-aged people, the amount of both accelerometer measured moderate to vigorous physical activity and self-reported LTPA were positively associated with PH regardless of daily time spent sitting. A clear dose-response relationship was observed, with both physical activity measures on PH and higher PA connected to better health. This study encourages use of both objective and subjective measurement of PA and the results can be utilized in policymaking and planning interventions and programs promoting physical activity at population level.

## Abbreviations

BMI: Body mass index
HRQoL: Health related quality of life
LTPA: Leisure time physical activity
MET: Metabolic equivalent of a task
MVPA: Moderate to vigorous physical activity
NFBC66: The Northern Finland Birth cohort 1966 study
OR: Odds ratio
PA: Physical activity
PH: Perceived health
SD: Standard deviation
ST: Sitting time
US: United States
